# Inhibition of influenza virus via a sesquiterpene fraction isolated from *Laggera pterodonta* by targeting the NF-κB and p38 pathways

**DOI:** 10.1186/s12906-016-1528-8

**Published:** 2017-01-06

**Authors:** YuTao Wang, Beixian Zhou, Jingguang Lu, QiaoLian Chen, Huihui Ti, WanYi Huang, Jing Li, ZiFeng Yang, Zhihong Jiang, XinHua Wang

**Affiliations:** 1 State Key Laboratory of Respiratory Disease, National Clinical Research Center for Respiratory Disease, First Affiliated Hospital of Guangzhou Medical University, Guangzhou, 510120 China; 2 State Key Laboratory of Quality Research in Chinese Medicine, Macau Institute for Applied Research in Medicine and Health, Macau University of Science and Technology, Taipa, Macau, SAR China

**Keywords:** *Laggera pterodonta*, Sesquiterpene fraction, Anti-influenza virus, Signalling pathway

## Abstract

**Background:**

Influenza virus poses serious threats to human health, especially human infection with avian influenza virus. *Laggera pterodonta* (DC.) Benth is a medicinal plant that is widely used in Traditional Chinese Medicine, especially in Yunnan province, and has been used to treat influenza, pharyngolaryngitis, and bronchitis. However, the compound(s) responsible for the activity and their mechanisms of action against the influenza virus remain to be elucidated.

**Methods:**

*L. pterodonta* extract was fractionated, and the active fraction was identified as Fraction 14 (Fr 14). Fr 14 was further analysed and characterized by ultra-high-performance liquid chromatography hyphenated with quadrupole-time of flight mass spectrometry (UHPLC/Q-TOF-MS). The inhibitory effect against influenza virus was evaluated using a cytotoxicity assay. Then, cytokines and chemokines were detected by qRT-PCR and a bio-plex assay. Signalling pathways that inhibited the influenza virus were identified using a western blotting assay.

**Results:**

The active fr 14 showed a wide spectrum of anti-influenza virus activity. The pharmacological mechanisms showed that Fr 14 acts on the early stage of virus replication (0–6 h). It inhibited the p38/MAPK pathway and then inhibited the NF-κB pathway and COX-2. Fr 14 also prevented the increased expression of cytokines and chemokines.

**Conclusion:**

This study demonstrated the preliminary mechanisms of fr 14 against the influenza virus. Fr 14 possessed antiviral and anti-inflammatory effects. *L. pterodonta* can be used to develop innovative antiviral drugs, and further studies will be performed to illustrate the detailed mechanisms.

## Background

Influenza viruses belong to the *Orthomyxoviridae* family and include types A, B, and C. Influenza A viruses have a wide spectrum of hosts and cause human respiratory infection, leading to severe annual morbidity and mortality. When the viruses undergo adaptive evolution, they can produce cross-species transmission between human and avian [1]. Recently, newly emerged influenza, such as H7N9, H10N8, and H5N6, have caused a severe threat to human health, especially the H7N9 virus, which has high rates of severe illness and death in patients [2].

To fight against these pathogens, some antiviral drugs have been developed and used in clinical practice, including oseltamivir, peramivir, zanamivir, amantadine, and rimantadine. Adamantine-derived drugs are not recommended due to drug resistance. All of the above antiviral drugs are resistant to influenza virus and are restricted to use in the clinic [3, 4].

Therefore, identifying and developing new antiviral drugs is urgently needed. *Laggera pterodonta* (DC.) Benth is a medicinal plant that is widely used in Traditional Chinese Medicine, especially in Yunnan province, and is used to treat influenza, pharyngolaryngitis, and bronchitis. A previous study showed that the flavonoids of *L. pterodonta* have anti-inflammatory and anti-apoptotic effects [5, 6]. Three dicaffeoylquinic acids isolated from *L. pterodonta* showed significant inhibitory activity against herpes simplex virus-1 (HSV-1), herpes simplex virus-2 (HSV-2) and influenza viruses A (IVA) in vitro [7].

To further study the pharmacological mechanism against influenza virus, the active fr 14 was isolated from *L. pterodonta*, and its chemical composition was analysed. Then, the antiviral spectrum and mechanisms were demonstrated in this study.

## Methods

### Plant medicine, cells and viruses

*L. pterodonta* was collected from Yunnan province. The herbarium specimen was authenticated by Professor Rongping Zhang and deposited in the College of Pharmaceutical Sciences, Kunming Medicine University. Madin-Darby canine kidney (MDCK) and A549 cells were purchased from the American Tissue Culture Collection (ATCC). The cells were grown in minimal essential medium (MEM) with 10% heat-inactivated foetal calf serum (FCS) supplemented with 1% penicillin and streptomycin. Oseltamivir carboxylic acid was purchased from TLC PharmaChem., Inc (Canada).

Influenza virus A/PR/8/34 (H1N1) and influenza virus A/Aichi/2/68 (H3N2) were purchased from ATCC while influenza (A/Guangzhou/GIRD/07/09, H1N1) and Flu B were isolated from routine clinical specimens. Several strains of avian influenza virus, including A/Duck/Guangdong/2009 (H6N2), A/Duck/Guangdong/1994 (H7N3) and A/Chicken/Guangdong/1996 (H9N2), were obtained from in-house repository. The influenza viruses were propagated in the allantoic cavities of chicken eggs.

### Isolation of a sesquiterpene fraction

The sample powder (40 g) was extracted using ultrasonic wave, adding 5 times methanol and repeating five times for 30 min. The extract was centrifuged at 2500 g for a further 10 min. The extracts were combined and condensed to a proper volume under reduced pressure. The solution was transferred to the MCI gel column and eluted with water, aqueous MeOH (10–100%) and methanol acetone (10–30%) of decreasing polarities to yield twenty-three fractions. The ultra-high-performance liquid chromatography hyphenated with mass spectrometry (UHPLC-MS) was used to analyze the fractions by comparing both accurate mass and fragment patterns. Fr 14 was found to be rich in sesquiterpenes.

### UHPLC/QTOF-MS analysis

Samples were analyzed on an Agilent 1290 Infinity UHPLC system (Santa Clara, CA, USA) equipped with a binary solvent delivery system and a standard auto-sampler. The conditions used were: column temperature 30 °C; injection volume 2.0 μl; mobile phase 0.1% aqueous solution formic acid (solution A) and acetonitrile (solution B). The mobile phase was programmed as follows: 0–8 min, solution B 45–70%; 8–10 min, solution B 70–100%. The mobile phase was pumped at a constant flow rate of 0.35 ml/min.

Mass spectrometry was performed using an Agilent 6540 ultrahigh definition (UHD) QTOF mass spectrometer (Santa Clara, CA, USA), equipped with a Jet Stream electrospray ionization (ESI) source. Parameters were as follows: Capillary voltage 4000 V for positive mode and 3500 V for negative mode, Nebulizer gas pressure 35 psi, drying gas flow rate 8 L/min, gas temperature 200 °C, nozzle voltage 300 V, skimmer 65 V, OCT RF V 600 V, fragmentor 150 V. The collision energy (CE) was set at 10V for MS mode and 10–40 V for auto MS/MS mode. The mass range recorded in the range of m/z 100–1700.

### Cytotoxicity assay

The cytotoxicity of various concentrations of fr 14 to MDCK cells were determined using an MTT assay. The cells, which were grown to 80–90% confluence in 96-well plates, were untreated or treated with the indicated amounts of drugs and cultured at 37 °C for 2 days. Then, the cells were treated with 5 mg/ml thiazole blue tetrazolium bromide in phosphate-buffered saline (PBS) and incubated for 4 h at 37 °C. The reaction product was dissolved in DMSO, and the cells were further incubated for 20 min at 37 °C. The absorbance was measured using a microplate reader at 570 nm [8]. The 50% toxic concentration (TC_50_) was calculated by Reed–Muench analysis [9].

### Inhibitory effect of fr 14

The 96-well plates were prepared and cultured with MDCK cells at 37 °C, 5% CO_2_ for 24 h. To evaluate the anti-influenza activity of the fraction, cells were washed with PBS and infected with 100 TCID_50_ (median tissue culture infective dose) of influenza virus (PR8 strain) at 37 °C for 2 h. Then, the medium was removed, and the indicated fractions were added at different concentrations with a two-fold dilution in serum-free MEM supplemented with 2 μg/ml TPCK-trypsin. After incubation for 48 h at 34 °C, the cytopathogenic efficiency (CPE) caused by the influenza virus was measured microscopically. The concentration required for 50% inhibition of the virus CPE (IC_50_) was calculated by the Reed–Muench method [10].

### Time of addition assay

MDCK cells growing in 24-well plates were then adsorbed with virus (A/PR/8/34, 0.01 MOI) for 2 h at 4 °C. Then, the cells were washed with cold PBS twice to remove the unbound virus. Next, MEM was added to the cells, and incubation was performed in a CO_2_ incubator at 37 °C. Fr 14 was added 2 h prior to the infection (-2 h) or at the same time with the virus infection (0 h), and at indicated time points post-infection (2 h, 4 h, 6 h, 8 h). Following incubation for 10 h, the supernatants were collected and infectious titres were determined by CPE assay [10].

### Detection of cytokines and chemokines by qRT-PCR

A549 cells growing in 96-well plates at 37 °C, 5% CO_2_ were prepared and then infected with influenza virus (A/PR/8/34, 0.1 MOI) for 2 h. The inoculums were removed, and the cells were treated with various concentrations of fr 14. The cells were collected at 24 h post-infection, and the total RNA was extracted using the TRIZOL reagent assay (Invitrogen) to detect the expression of TNF-*α*, IL-8, IL-6, MCP-1, IP-10, and RANTES by quantitative RT-PCR using the ABI 7500 Real-time PCR System [11].

### Detection of cytokines and chemokines by bio-plex assay

A549 cells were grown in 6-well plates and then washed with PBS twice. The virus (A/PR/8/34, 0.01 MOI) was incubated with the cells for 2 h. Then, fr 14 was added at different concentrations. The supernatants were collected after 24 h and centrifuged at 13000 rpm at 4 °C to remove the cell debris. Cytokines were detected using the bio-plex liquid phase chips kit with the bio-plex 200 system [11].

### Western blotting assay

A549 cells were prepared and washed with PBS, then incubated with virus A/PR/8/34 (MOI = 0.1) diluted in PBS for 30 min at 37 °C. Then, the inoculums were discarded, and the cells were incubated with MEM in the absence and presence of different concentrations of fr 14 for 24 h at 37 °C. Cell lysis and western blots were performed as previously described [10].

## Results

### Characterization of fr 14

**Fig. 1:**
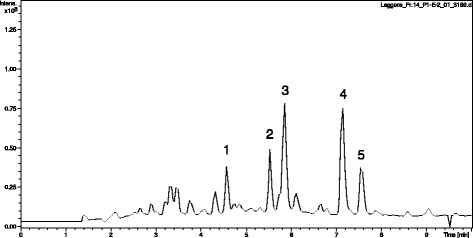
UHPLC-QTOF MS total ion chromatogram of the sesquiterpene fraction obtained from *L. pterodonta*

**Fig. 2:**
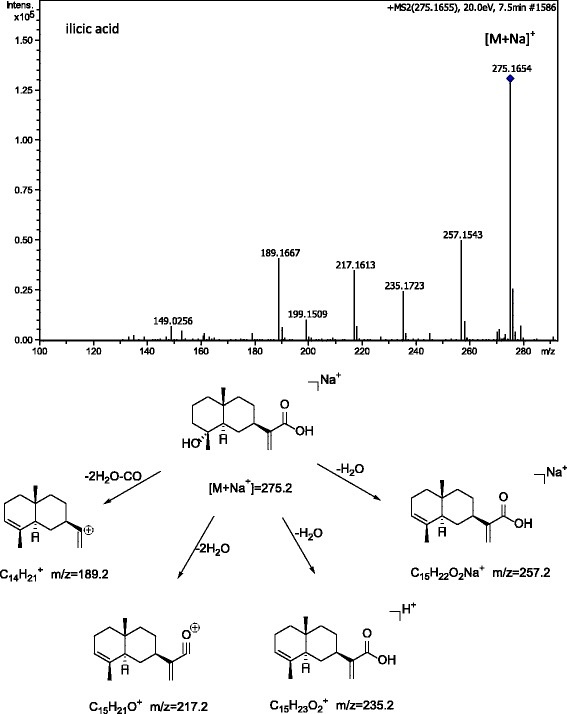
MS/MS spectrum illustration for compound of peak 5 in positive mode

The chemical data of the proposed compounds are shown in Table 1. Here we take peak 5 in Fig. 1 as an example to illustrate its identification process. Precursor ions of peak 5 were obtained in positive mode and negative mode, offering molecular fomular of C_15_H_24_O_3_. Further more, MS/MS fragments were observed selecting *m/z* 275.16 ([M + Na]^+^) as precursor ion. Compound of peak 5 was illustrated as shown in Fig. 2, and it was identified as compound of ilicic acid by comparing accurate mass and molecular formula with data reported in the literature [12]_._ Besides, precursor ions and MS/MS fragments from peak 2 were found almost the same as those of peak 5, which indicated difference is the position of hydroxyl group between compounds of peak 2 and peak 5. Therefore, compound of peak 2 was identified as an isomer of ilicic acid. By using the similar procedure, other compounds could be identified in this experiment [13].

**Table 1:** The chemical data of fr 14

Number. peak	Formula	Compound name	Reference
1	C_15_H_20_O_3_	Tessaric acid or pterodonoic acid	[26, 27]
2	C_15_H_24_O_3_	Isomer of ilicic acid	[12]
3	C_15_H_20_O_3_	Tessaric acid or pterodonoic acid	[26, 27]
4	C_15_H_22_O_3_	2α-Hydroxypterodontic acid; or 1β-Hydroxypterodontic acid; or 3β-Hydroxypterodontic acid; or 5α-Hydroxylcostic acid; or 5β-Hydroxylcostic acid	[26, 28, 29]
5	C_15_H_24_O_3_	Ilicic acid	[12]

### Antiviral spectrum of fr 14

The antiviral spectrum determined by CPE assay demonstrated that fr 14 can inhibit different influenza strains, namely influenza viruses A/PR/8/34, A/Guangzhou/GIRD07/09 (H1N1) and A/Aichi/2/68 (H3N2), but failed to inhibit avian influenza virus, such as H6N2, H7N3, and H9N2 (Table 2).

**Table 2:** The antiviral spectrum of fr 14

Virus strains	fr14 (μg/ml)			Oseltamivir (μg/ml)		
	TC_50_	IC_50_	SI	TC_50_	IC_50_	SI
A/PR/8/34 (H1N1)	>200	79.4	>2.52	>1000	0.05	>1000
A/Guangzhou/GIRD07/09 (H1N1)	>200	43.5	>4.59	>1000	0.11	>1000
A/Aichi/2/68 (H3N2)	>200	75	>2.67	>1000	0.06	>1000
Flu B	>200	>100	<2	>1000	6.31	>150
A/Duck/Guangdong/2009 (H6N2)	>200	>150	<1.33	>1000	NT^a^	NT^a^
A/Duck/Guangdong/1994 (H7N3)	>200	>150	<1.33	>1000	NT^a^	NT^a^
A/Chicken/Guangdong/1996 (H9N2)	>200	>150	<1.33	>1000	NT^a^	NT^a^

^a^NT: not test

### The inhibition stage of influenza virus replication by fr 14

**Fig. 3:**
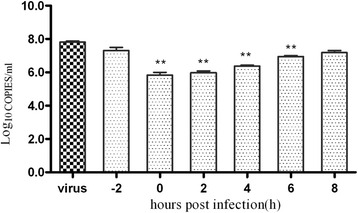
The time-of-addition assay. MDCK cells were prepared in a 24-well plate and then adsorbed with virus (A/PR/8/34, 0.01 MOI) for 2 h at 4 °C. Then, the cells were washed and MEM was added to the cells. Fr 14 was added 2 h prior to the infection (-2 h) or at the same time as the virus infection (0 h) and at the indicated time points post-infection (2 h, 4 h, 6 h, 8 h). Following incubation for 10 h, the supernatants were collected, and infectious titres were determined by CPE assay

A time-of-addition experiment was performed to confirm the stage of influenza virus replication influenced by fr 14. Fr 14 was added at different time points and showed potent antiviral activity at 0–6 h, which was during the early stage of virus replication (Fig. 3). Therefore, the entry and absorption step, or endosomal, of nucleic acid release of the influenza virus might have been inhibited by fr 14.

### Inhibition of inflammatory cytokines in A549 cells post influenza virus infection in mRNA and protein levels

**Fig. 4:**
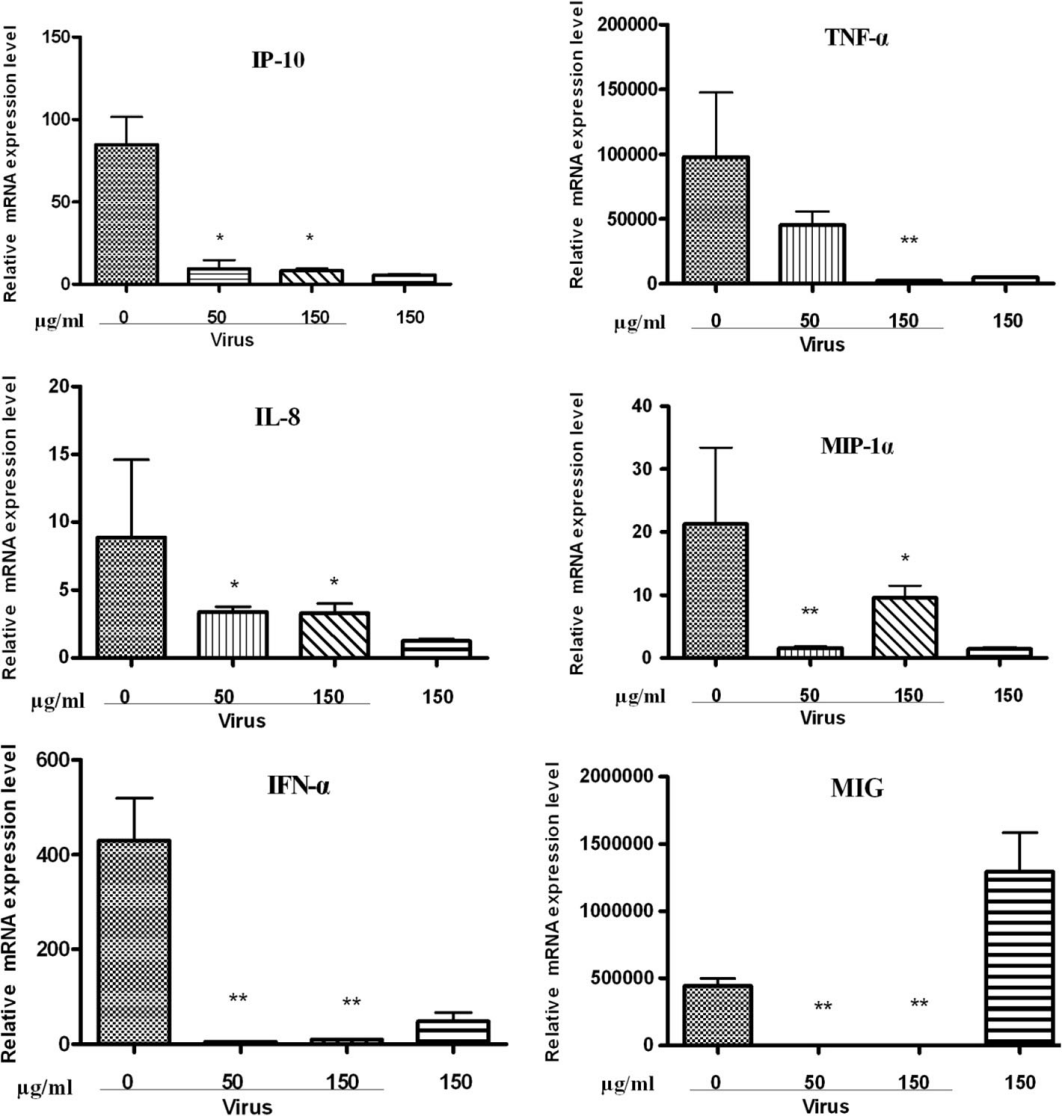
Inhibition of inflammatory cytokines in A549 cells post influenza virus infection at the mRNA level. A549 cells growing in a 96-well plate at 37 °C, 5% CO_2_ were prepared and then infected with influenza virus (A/PR/8/34, 0.1 MOI) for 2 h. The inoculums were removed, and the cells were treated with various concentrations of fr 14. The cells were collected at 24 h post-infection to determine the expression of IP-10, TNF-*α*, IL-8, MIP-1*α*, IFN- *α*, and MIG by RT-PCR

Inflammatory cytokine expression deregulation is a risk factor for the healthy population, and excessive secretion will cause tissue damage. Therefore, the effects of fr 14 on inducing cytokine productions were determined. The results showed that the mRNAs of IP-10, TNF-*α*, IL-8, MIP-1*α*, IFN- *α*, and MIG were significantly reduced in fr 14-treated cells at 24 h post infection (Fig. 4).

**Fig. 5:**
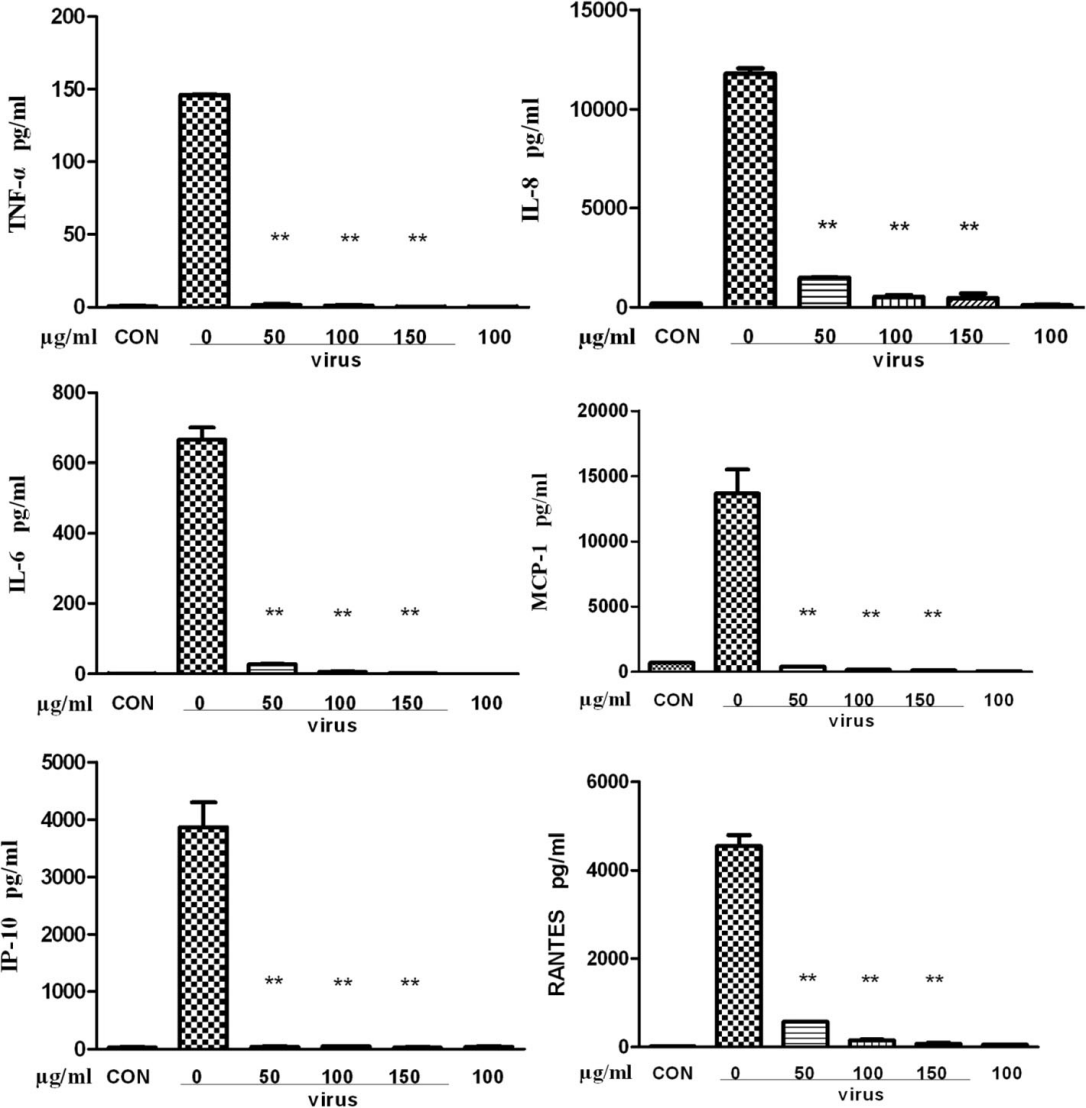
Inhibition of inflammatory cytokines in A549 cells post influenza virus infection at the protein level. A549 cells were grown in a 6-well plate and then washed with PBS twice. The virus (A/PR/8/34, 0.01 MOI) was incubated with the cells for 2 h, and fr 14 was added at different concentrations. The supernatants were collected after 24 h, and the cytokines were detected using the bio-plex liquid phase chips kit with the bio-plex 200 system

**Fig. 6:**
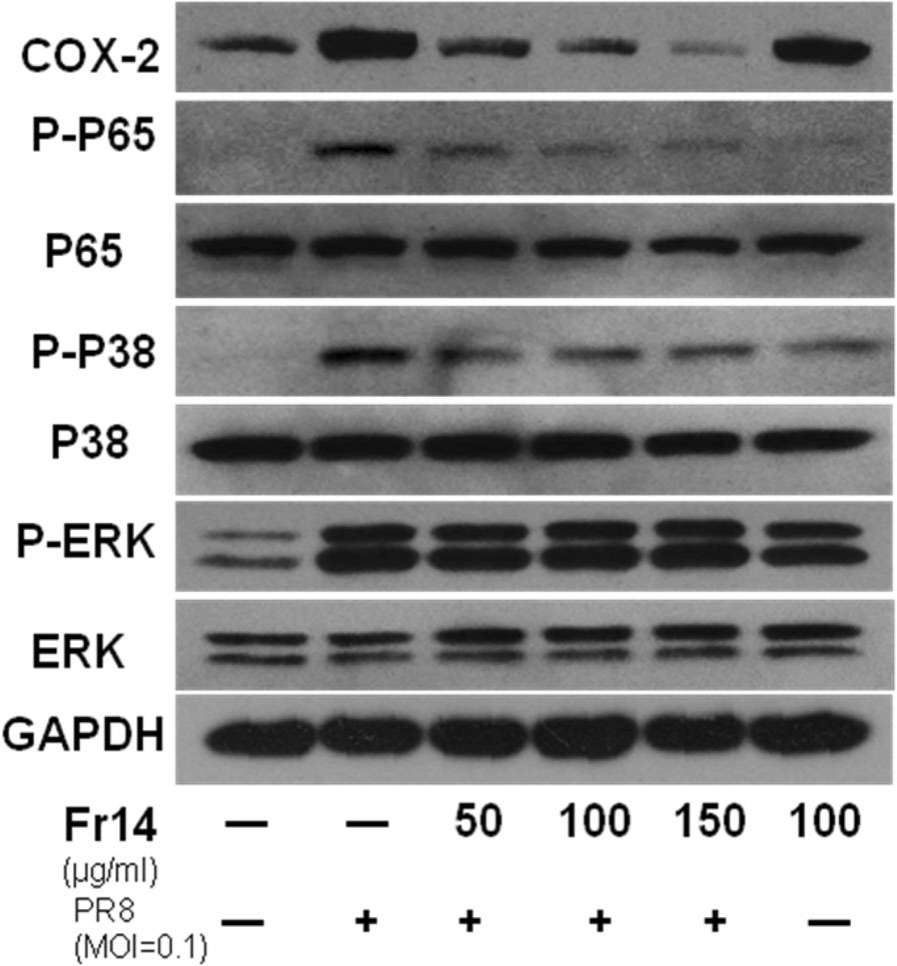
Inhibition of influenza virus-induced signalling pathway by fr14. A549 cells were washed with PBS and subsequently incubated with virus A/PR/8/34 (MOI = 0.1) diluted in PBS for 30 min at 37 °C. Then, the inoculums were aspirated, and the cells were incubated with MEM in the absence and presence of different concentrations (150, 100, 50 μg/ml) of fr 14 for 24 h at 37 °C. Cell lysis and western blots were performed

The inflammatory cytokines were further verified at protein levels. The bio-plex analysis results showed that higher levels of TNF-*α*, IL-8, IL-6, MCP-1, IP-10, and RANTES expression were detected in the influenza virus infection group but that the levels in the drug-treated group of different concentrations (150, 100, 50 μg/ml) decreased (Figs. 5 and 6).

### Inhibition of influenza virus-induced signalling pathway by fr14 

Influenza virus infections cause NF-κB, p38, and ERK activation. The results in Fig. 5 showed that fr 14 can inhibit the protein phosphorylation of p65 and p38, but had no effect on the ERK pathway. NF-κB plays an important role in the regulation of COX-2 and iNOS expression; therefore, COX-2 was also inhibited.

## Discussion

The influenza virus causes respiratory disease, including headache, cough, sore throat, rhinorrhoea, and severe pneumonia, and these symptoms are especially prevalent in newly emerged viruses, such as recombinant avian influenza virus [14, 15]. However, anti-influenza drugs are limited due to resistance. Therefore, it is necessary to explore alternative drugs.

Traditional Chinese medicines (TCM) have been used for a long time clinically. Various extracts, fractions or compounds isolated from TCM have demonstrated antiviral activity with different mechanisms [16].

In our research, fr 14 was isolated from *L. pterodonta*, and the antiviral mechanisms of fr 14 were detected in our present study. First, the specific steps of influenza virus replication inhibited by fr 14 were tested. The results showed that the fraction can inhibit virus replication when added at 0–6 h, which indicates that the entry and absorption step, or endosomal, nucleic acid release, may be inhibited. Another possibility is related to the host immune regulation.

Influenza viruses are parasitic and need the host’s cellular function to achieve their life cycle. The previous study showed that 219 of the 295 factors were required for efficient influenza virus growth based on a genome-wide RNAi screening experiment [17].

Influenza virus infection can induce the host signalling pathways, such as ERK/MAPK, p38/MAPK, and NF-κB. ERK and p38 belong to the mitogen-activated protein kinase (MAPK) family, which is involved in cell growth, apoptosis and the immune response [18]. Raf/MEK/ERK is essential for influenza virus replication, and the ERK inhibitor U0126 can inhibit the nuclear export of the viral RNPs in the virus replication cycle [19].

The nuclear factor kappa B (NF-κB) families are critical transcription factors regulating inflammation and apoptosis. The influenza virus proteins HA and NA, or viral RNA accumulation, can activate the NF-κB signalling pathway, which is the hallmark of virus infection. It was reported that the NF-κB signalling pathway can be exploited by the virus to block apoptosis and prolong the survival of the host cell and increase viral progeny production. NF-κB is also involved in the inflammatory response, inducing the transcription of proinflammatory cytokines, such as TNF-α, IL-6, and IL-8, and the expression of enzymes, such as inducible cyclooxygenase [20, 21]. P38 is also related to the innate immune response, controlling the expression of cytokines such as RANTES, IL-8 and TNF-α [22], and research has demonstrated that p38/MAPK may act as an upstream activator of the NF-κB signalling pathway [23]. Our results indicated that fr 14 inhibited the protein p65/NF-κB and p38/MAPK phosphorylation at concentrations of 50, 100, and 150 μg/ml but did not affect ERK/MAPK. Based on previous data [23], the mechanisms of fr 14 may be a cascade process that inhibited p38/MAPK, inhibited NF-κB, and further inhibited COX-2.

Cytokines and chemokines play an important role in influenza virus infection, especially highly pathogenic influenza viruses. TNF-*α*, IFN-*α*, and IL-1 are expressed in the early cytokine cascade, followed by IL-6 and chemokines, such as IL-8, MCP-1, MIP-1, IP-10, and MIG [24]. Previous findings found that IL-6 may be a potential disease severity biomarker for severe pandemic H1N1 Influenza A infection [25]. Chemokines IP-10 and RANTES can act to damage host tissue by recruiting monocytes, macrophages, DCs, and T cells that enhance inflammatory processes [24].

Our results demonstrated that TNF-*α*, IL-8, IP-10, MIG, MIP-1*α*, and IFN-*α* were decreased at the mRNA level after treatment by fr 14 (50, 150 μg/ml). The bio-plex analysis results demonstrated that the drug-treated group of different concentrations (150, 100, 50 μg/ml) can inhibit TNF-*α*, IL-8, IP-10, IL-6, MCP-1, and RANTES expression at the protein level. The inhibition of increased cytokine and chemokine expression may also be related to the p38 and NF-κB pathways.

## Conclusion

In this study, active fractions were isolated from *L. pterodonta.* Fr 14 had a wide spectrum of anti-influenza virus activity. The pharmacological mechanisms showed that fr 14 acts on the early stages of virus replication (0–6 h). Fr 14 inhibited p38/MAPK and then inhibited NF-κB and COX-2. Fr 14 also prevented an increase in cytokines and chemokines expression. *L. pterodonta* can be used to develop an innovative antiviral drug, and further studies will be performed to illustrate the detailed mechanisms.

## Abbreviations

ATCC: American Tissue Culture Collection
CE: Collision energy
CPE: Cytopathogenic efficiency
FCS: Foetal calf serum
HSV: Herpes simplex virus
IVA: Influenza viruses A
MDCK: Madin-Darby canine kidney
MEM: Minimal essential medium
PBS: Phosphate-buffered saline
SI: Selective index
TCID_50_: 50% median tissue culture infective dose
